# Multi-Transcripts and Expressions of *Trypsin Inhibitor* and *α-Amylase Inhibitor* Genes of Sengon (*Falcataria falcata*) Against *Xystrocera festiva* Stem Borer Infestation

**DOI:** 10.3390/plants14172750

**Published:** 2025-09-03

**Authors:** Ulfah Juniarti Siregar, Hasyyati Shabrina, Esti Nurianti, Fahirah Dwiyuni, Ayu Indah Lestari, Januard Kristian Sihombing, Buma Larosa, Vilda Puji Dini Anita, Deden Derajat Matra

**Affiliations:** 1 Department of Silviculture, Faculty of Forestry and Environment, IPB University, Bogor 16680, West Java, Indonesia; ulfahjs@apps.ipb.ac.id (U.J.S.); esti.nurianti2@gmail.com (E.N.); fahirah34@gmail.com (F.D.); aaayuindahlestari@gmail.com (A.I.L.); januard.sihombing@gmail.com (J.K.S.); bumalarosa2@gmail.com (B.L.); 2 Forestry Program, Faculty of Agriculture University of Mataram, Jalan Pendidikan No 37, Mataram City 83125, West Nusa Tenggara, Indonesia; 3 Department of Forestry Engineering, Faculty of Industrial Technology, Institut Teknologi Sumatera, Bandar Lampung 35365, Lampung, Indonesia; vilda.anita@rh.itera.ac.id; 4 Department of Agronomy and Horticulture, Faculty of Agriculture, IPB University, Bogor 16680, West Java, Indonesia

**Keywords:** *Falcataria falcata*, gene expression, *trypsin inhibitor*, *Xystrocera festiva*, *α-amylase inhibitor*

## Abstract

The infestation of boktor (*Xystrocera festiva* Pascoe) stem borer in Sengon (*Falcataria falcata*) tree plantations in Indonesia, especially in Java, has caused severe losses by damaging the stems, decreasing wood quality, and potentially leading to mortality. To digest the woods, the *X. festiva* larvae’s gut has at least two digestive enzymes, which are trypsin and α-amylase. Former studies have shown that *F. falcata* produces inhibitors of these two enzymes as part of its defense mechanisms. This research aimed to analyze *trypsin inhibitor (TI)* and *α-amylase inhibitor (AAI)* transcripts, as well as their expression, in *X. festiva*-infested and resistant *F. falcata* trees. We found 19 contigs encoding the *TI* gene and 29 contigs encoding *AAI.* The results were able to predict the sequence of the DNA that produced the *TI* and *AAI* transcriptomes, which proved that one gene could be expressed differently due to alternative splicing. The DEG analysis and RT-PCR confirmed that mostly the *TI* and *AAI* activity was heavily induced by the *X. festiva* larvae attacks. The expression of the *TI* gene was upregulated 0.78 times, while the *AAI* gene expression was upregulated 2.44 times in infested samples. The findings from this study are fundamental in understanding the mechanism of *F. falcata* resistance against *X. festiva* infestation and selecting the resistant trees.

## 1. Introduction

*Falcataria falcata*, commonly known as albizia, Jeungjing (Sundanese), Batai (Malaysian), Bae (Papuan), or Sengon (Indonesian), is a tree species native to Haiti, Papua New Guinea, the Solomon Islands, and Indonesia [1]. It is valued for its fast growth characteristics and fast economic return in Indonesia [2], especially on Java Island. It is the most common crop commodity planted in community forests in Java, which produces 97.16% of total *F. falcata* production in Indonesia [3]. The *F. falcata* wood industry itself is growing rapidly and has started to enter the international market.

With the rapid growth of the industry and plantation size, especially in monoculture form, the threat to the plantation itself has also increased. There is one major pest of *F. falcata*, the boktor stem borer (*Xystrocera festiva*), which starts infestation when the plant reaches 3 years old [4]. The damage is caused mainly by the larval stage of the insect. The insect infestation begins when the eggs are laid in bark crevices or wounds on the tree. The hitched larvae then create feeding tunnels or burrowing holes that run downwards and become larger as the larvae’ size increases as well [5]. The feeding tunnels decrease the quality and quantity of timber, damaging the stems, and leading to plant mortality [6]. The infestation itself is controlled mainly by eradicating the infested tree, but the spread of *X. festiva* infestation still happens widely and is deadly.

*X. festiva* belongs to the family Cerambycidae in the Coleoptera order, in which the larvae are major wood borers in forest areas [7]. The larvae mainly feed on the starch of the wood [8], and also need protein for survival, growth, and fecundity [9]. To digest those two main nutrients, the larvae’s guts have enzymes to break down the complex molecules into smaller ones. One of the important enzymes to digest carbs in the *X. festiva* digestive system is *α*-amylase, which acts in the first step of breaking starch down in the form of maltopolysaccharides [10] into oligosaccharides [11]. Meanwhile, to digest the proteins, the larvae secrete serine protease enzymes, including the major enzyme trypsin. Trypsin is involved in the initial phase of protein digestion by cleaving the bonds of the polypeptide on the carboxyl side of basic L-amino acids, lysine, or arginine [12].

To suppress the *X. festiva* larvae’s feeding ability, *F. falcata* trees secrete inhibitors of those digestive enzymes [13], which interfere with the digestion of plant starch and proteins. Amylases play a role in softening the cellulose in the wood so it will be easier to digest [14], while trypsin breaks the protein chain on the carboxyl side of basic L-amino acids [15]. Two known inhibitors produced by the *F. falcata* tree were *trypsin inhibitor* (TI) and *α-amylase inhibitor* (AAI), which inhibit trypsin and α-amylase secreted by herbivores to digest proteins, starches, and glycogens in plants [9,16]. A former study stated that TI and AAI activity had negative correlations with the larvae’s weight, length, and feed consumption in an artificial diet [13]. Other studies in the standing *F. falcata* trees showed that TI and AAI activities were higher in resistant trees’ woods and barks [17,18]. Studies of TI and AAI in other plants have also shown that those two inhibitors play a significant role in plant protection against herbivores [19,20,21,22].

The current advancement in technologies has enabled us to analyze the expression of genes encoding those two enzymes in the transcriptomes produced from the infested and resistant trees. Large-scale genotyping, genomic sequencing, and gene analysis have enabled us to have a better and more comprehensive understanding of plant–herbivore interactions and identify the key resistance components that may be integrated in future tree improvement. Ribonuclease acid–sequencing (RNA-seq) is now recognized as the most powerful, robust, and adaptable technique for measuring gene expression and transcriptional activation at the genome-wide level. Other research has shown that various resistance genes, including *AAIs*, were expressed in pigeon pea against the pod-borer using RNA-Seq analysis [23]. Another study found various peptidase inhibitors in the banana transcriptome against the pseudostem weevil [24]. This research objective was to analyze the difference in the RNA-seq from *X. festiva*-infested and resistant *F. falcata* trees at the transcriptome level and identify the *TI* and *AAI* gene expression. Findings from this research will be significant in developing markers for selecting the resistant *F. falcata* trees.

## 2. Results

### 2.1. Contigs Exploration and Phylogenetic Analysis

**Figure 1:**
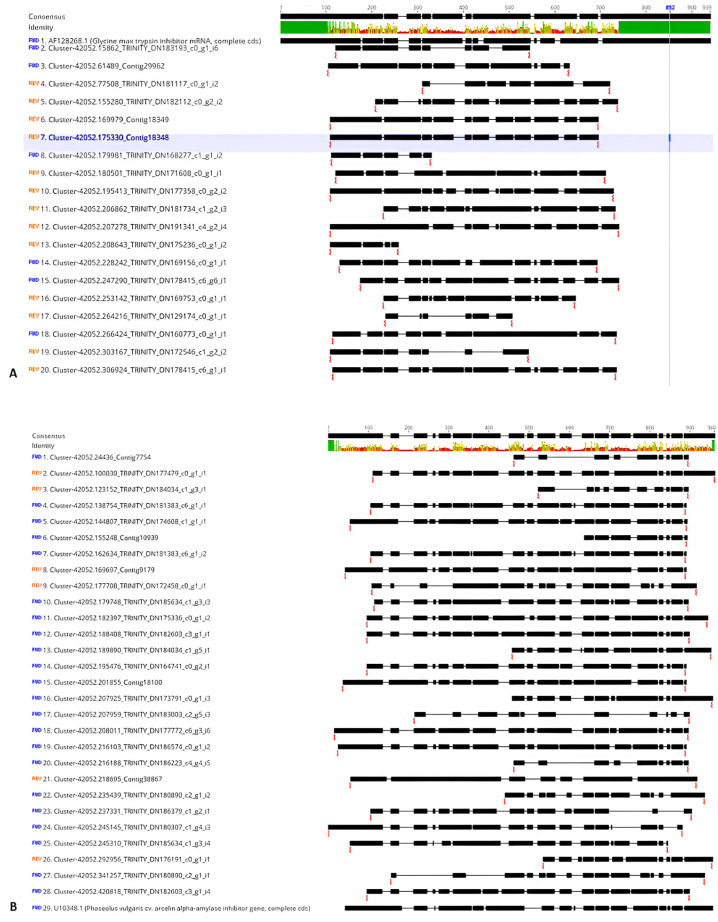
The alignment of *TI*-containing contigs from *Falcataria falcata* transcripts. With *Glycine max TI* (accession number AF128268.1) (**A**) and *AAI*-containing contigs with *Phaseolus vulgaris AAI* (accession number U10348.1) (**B**).

The assembled sequences of the samples and the properties are available at https://doi.org/10.6084/m9.figshare.14058458 [25]. The annotated contigs were blasted with reference sequences and resulted in 19 contigs of *TI* and 29 contigs of *AAI* found in all the transcriptomes (Table 1). The *AAI*-containing contigs of *F. falcata* length were 201–728 base pairs (bp); meanwhile, the *TI*-containing contigs length range was 125–764 bp. The *TI* contigs BLAST v.2.14.1 results showed that all the contigs were homologous with Kunitz-type *trypsin inhibitor* from various members of the Fabaceae family. The alignment of *TI*- and *AAI*-containing contigs is shown in Figure 1. We used the *TI* sequence from *Glycine max* and the *AAI* sequence from *Phaseolus vulgaris* as a comparison, since they belonged to the same family. The aligned contigs from *F. falcata* had identical length and gaps with the *TI* sequence from *G. max*, and *AAI* from *P. vulgaris*. The consensus length for *TI* alignment was 927 bp, and for *AAI* was 1061 bp. A consensus is built from the most common residues at each position (alignment column) such that the total percentage of rows represented by the selected residues in that column meets at least the specified threshold. The alignment of the contigs in Figure 1 showed that there were sites that aligned with reference sequences from *G. max* and *P. vulgaris*. Similar lengths and gaps indicated that there was high similarity between all aligned contigs.

The alignment of *TI*- and *AAI*-containing contigs is shown in Figure 1. We used the *TI* sequence from *G. max* and the *AAI* sequence from *P. vulgaris* as a comparison. It can be seen that there is an identical length and gaps between aligned contigs. Deep gaps in alignment indicate change mutations in sequences, including insertion, deletion, or rearrangement of nucleotides.

The phylogenetic trees from *TI* and *AAI* showed that there were no clustered contigs. All of the contigs were clustered into one big group against the outgroup sequence. The importance of the outgroup in generating a phylogenetic tree was to create character polarization. The outgroup for *TI* was chosen because in a former study, there was a 24 kilodalton (kDa) protein from *F. falcata*’s bark and wood, which was identical to *TI* from *G. max*. Meanwhile, *AAI* from *P. vulgaris* was chosen as the outgroup because it also belonged to the lectin group of *AAI*. All of the *TI*-containing contigs from our study were from Kunitz-type TI. There were two major groups formed in the *TI* phylogenetic tree, which were on the same line (not branched) as the outgroup one. In Group II, there was further branching so that the members were not in the same branch as the outgroup. Group 1, which is in the same line, has a closer kinship with the outgroup (AF128268.1) and had a genetic distance of 1.329. Overall, Group II, whose members were more diverse and were not in the same branch as the outgroup sequence, had a genetic distance value of 1.322. The value of genetic distance in Group II’s *F. falcata* contigs did not differ much from Group I’s contigs due to the branching between the two groups, with the outgroup starting at the same beginning. The difference between the two groups was due to the location and length of the nucleotide base sequences in the different conserved domains.

**Figure 2:**
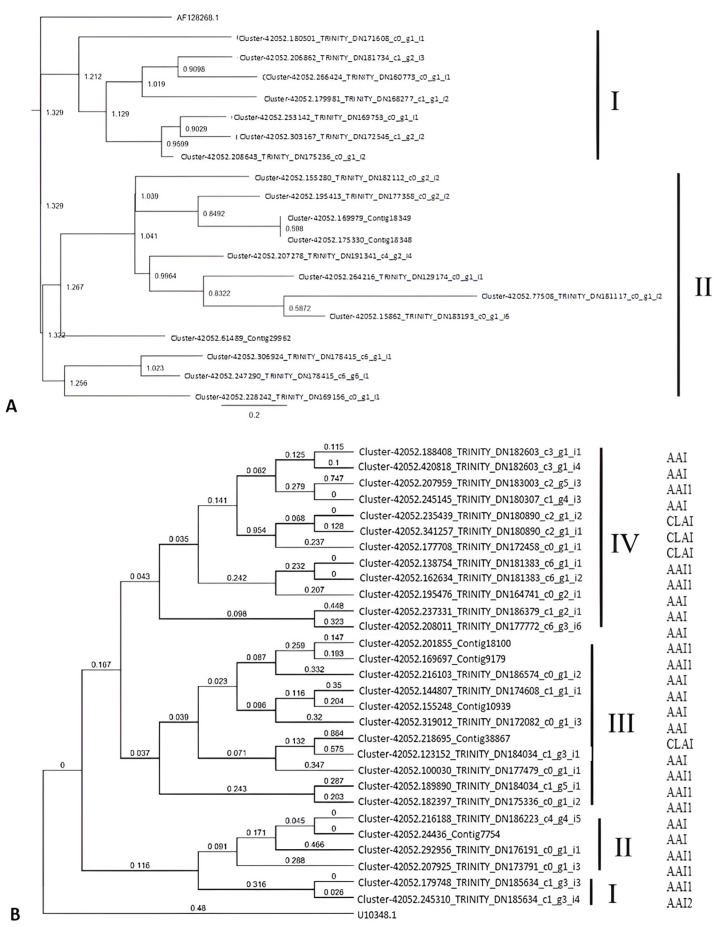
Phylogenetic tree of *TI*-containing contigs from *F. falcata* transcripts with *G. max TI* (accession number AF128268.1) (**A**) and AAI-containing contigs with *P. vulgaris* AAI (accession number U10348.1) (**B**).

Phylogenetic analysis in *AAI* contigs showed the presence of four groups that belonged to the lectin class. Group differences were due to differences in splicing sites for both exons and introns. Group I consisted of two contigs that were identified as AAI1 and AAI2, which inhibit lectins and alpha-amylase and act as a defense protein against insects. Group II consisted of four contigs that were identified as AAI and AAI1. Group III consisted of 11 contigs, and Group IV consisted of 12 contigs. Groups III and IV were identified as AAI, AAI1, and CLAI (a legumin-amylase inhibitor from Cicer arietinum). Phylogenetic analysis results showed that Groups III and IV evolved faster than Groups I and II because of the higher number of divergences. Phylogenetic analysis showed that Groups III and IV had a higher number of branches than Groups I and II, and a lower identical site value of 2% and 3.4%. The phylogenetic trees of *TI* and *AAI* are shown in Figure 2. Meanwhile, it was suggested that AAI2 evolved from AAI1 because of the high homology between both contigs. This hypothesis concurred with the results of phylogenetic analysis, with AAI1 and AAI2 contigs in this study belonging to the same group with 95.3% identical site values.

### 2.2. Comparison of Transcriptomes and Genome Draft

**Figure 3:**
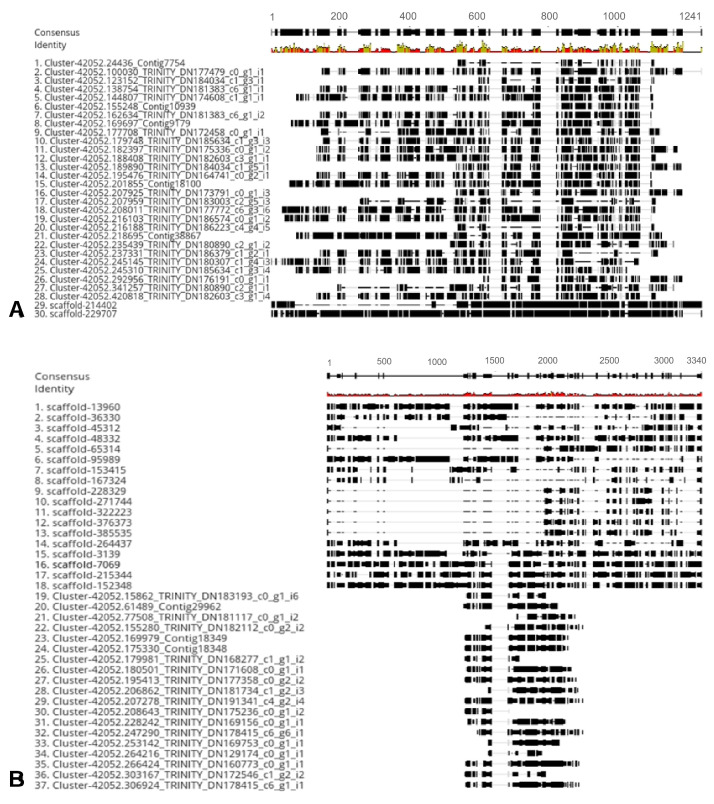
The spliced alignment of *AAI* (**A**) and *TI* (**B**). Contigs: transcriptome sequence; scaffolds: genome draft sequence.

Genome sequencing produced 72.6 GB of raw data, which was deposited at DDBJ with accession number DRA012508 (https://ddbj.nig.ac.jp/search/entry/sra-submission/DRA012508, accessed on 31 July 2021). The assembling of the genome using Ray software v.3 produced 1,074,927 sequences, with an N50 value of 710 bp and a maximum length of 133,812 bp. The annotation process resulted in 70.09% of the sequences matching with proteins from the NR NCBI, and 11.05% matched with proteins from UniProt. We found two candidates of genome sequences that contained *AAI* and 18 sequences that contained *TI* genes. The genome sequences aligned with the transcriptomes and resulted in the predicted genome sequence that transcribes the *AAI* and *TI* genes. The spliced alignments of *AAI* and *TI* are shown in Figure 3A,B.

Based on the results from the spliced alignment of *AAI* and *TI,* we predicted that the *AAI* genes were produced by scaffold-229707, while for *TI* genes, we found two candidates, which were scaffold-152348 and scaffold-215344.

### 2.3. Differentially Expressed Gene Analysis and Real Time-Polymerase Chain Reaction

**Figure 4:**
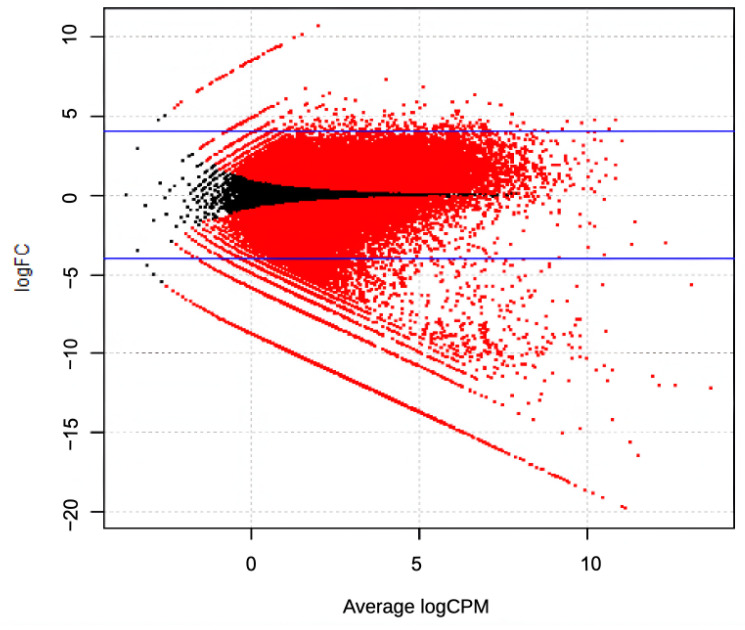
DEG results of the resistant and infested *F. falcata* trees.

The assembled contigs showed that from the total of 10,566 contigs, 6859 were expressed in an upregulated way, and 3707 were expressed in a downregulated way (Figure 4).

**Figure 5:**
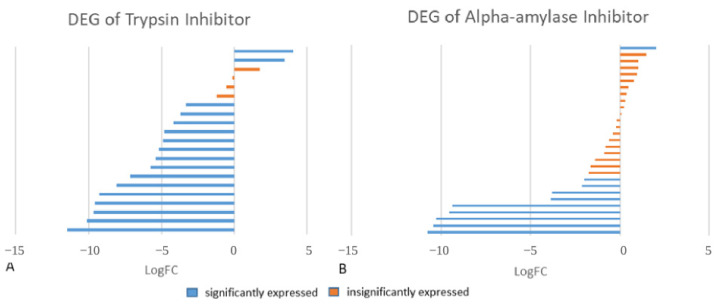
The logFC value of *TI* (**A**) and *AAI* (**B**) contigs in DEG analysis from RNA-Seq of *F. falcata*.

The DEG analysis between resistant and infested samples showed that mostly *TI* and *AAI* in *F. falcata* were expressed in a downregulated manner in susceptible trees. The logFC of *TI* ranged from −11.472 to 4.027, and *AAI* logFC ranged from −10.752 to 2.016 (Figure 4). The DEG results showed that the genes were expressed variously, with 9.52% of the *TI* expressed in an upregulated way, 71.43% expressed in a downregulated way, and 19.05% expressed in an insignificant way. For *AAI*, the DEG showed that the isoforms of the gene were also expressed in both an upregulated (3.45%) and downregulated (31.02%) way, while 65.52% was expressed in an insignificant way (LogFC < 2). The DEG analysis results for *TI* and *AAI* genes found in the transcriptome are shown in Table 2 and Figure 5. These findings indicated that somehow the *TI* and *AAI* expression increased in the stem after the larval infestation. The results were then confirmed using RT-PCR.

**Figure 6:**
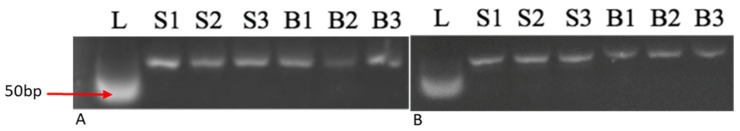
The amplicons amplified by *TI* (**A**) and *AAI* (**B**) primers with 50 bp ladder (L). S1–S3: samples from resistant trees 1–3; B1–B3: samples from *X. festiva*-infested trees 1–3.

From all the transcriptomes, the number of primers that were available for primer designing was seven for *TI* and seven for *AAI*. Out of 14 pairs of primers generated, only one pair from each *TI* and *AAI* produced specific results and had an appropriate melt curve with a single peak. The primers that were able to produce a specific band in this study are listed in Table 3. The desired single peak in the melting curve showed that our amplified product was specific, and the PCR process produced single-band results. The RT-PCR products were also electrophoresed in 2% agarose to ensure the size of the PCR products. The electrophoresis results are shown in Figure 6.

**Figure 7:**
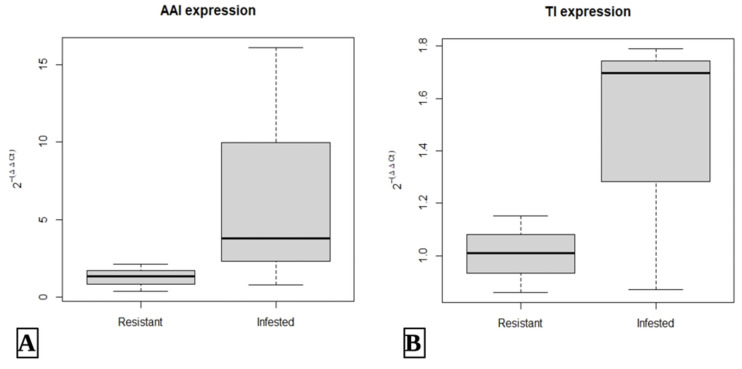
Relative expression of *AAI* (**A**) and *TI* (**B**) genes in *F. falcata* against *X. festiva*.

The RT-PCR data showed that the expression of the *TI* gene was already high (average 0.860) and increased after the infestation (average 1.790). On the other hand, *AAI* expressions were considered low (average 1.262) and highly increased after infestation (average 6.899). These findings indicate that both genes were already present as *F. falcata* defenses against *X. festiva* larvae, but the expression of both genes increased due to the induction of larval trypsin and α-amylase infestation. These results were in accord with the DEG analysis results from RNA-Seq. The results showed that in the infested samples, expression of the *TI* gene was upregulated 0.78 times compared to the resistant ones, while for the *AAI* gene, the expression was upregulated 2.44 times in infested samples. The expression difference of *AAI* was significant with a *p*-value of 0.04 but considered insignificant in the *TI* gene with a *p*-value of 0.45. The complete results of the RT-PCR are shown in Figure 7 below.

## 3. Discussion

The results from our study showed that there were several isoforms of *TI* and *AAI* as a result of different assembly of the transcriptomes from RNA-Seq using NGS technology. Most eukaryotic genes have multiple isoforms. Isoforms of mature RNA (mRNA) derived from the same locus are molecules with different exon compositions and lengths and can encode different forms of the corresponding genes [26]. The isoforms happened because all the protein-coding genes were separated by introns that have to be removed through splicing in mRNA maturation [27]. Splicing is carried out by spliceosomes, large ribonucleoprotein (RNP) complexes found in eukaryotes that assemble around splice sites in introns of pre-mRNA molecules and catalytically remove introns through successive reactions of phosphodiester transfer [28]. There were a large number of proteins that act as splicing regulators, which recognized distinct sequences in RNA as ‘splicing code’ [29]. When the mRNA sequences were constitutively spliced, the exons were joined in an order that corresponds to the DNA following the removal of the introns. When the mRNA sequences were constitutively spliced, the exons were joined in an order that corresponds to the DNA following the removal of the introns.

Many identical copies of RNA can be made from the same DNA template, and each RNA molecule can direct the synthesis of many identical protein molecules, allowing cells to rapidly synthesize large amounts of protein on demand [30]. In this study, we used a splice alignment method, which aligned the predicted transcriptomes to the predicted genome sequences [31]. The alignment of transcriptomes and genome draft from our study showed that the *AAI* and *TI* genes most probably originated from a single copy of DNA [30]. Alternative splicing deviates from this process through mechanisms that rearrange exon patterns into different coding sequences that are translated into different proteins [32]. Alternative splicing is a post-transcriptional level of gene expression regulation that increases the diversity of transcriptome and proteome. Alternative splicing happened because of the occurrence of alternate-splice sites in the premature RN [33]. The alternative splicing-related RNA-binding proteins (RBPs), under some conditions, could lead to different splicing events [34]. The isoforms were formed by assembling the spliced reads and mapping them to the reference transcriptome, then clustered and filtered to obtain high-quality contigs [35]. De novo assembly is the sole method available to reconstruct transcriptional isoforms from short-read methods in organisms for which there was not a high-quality reference genome, yet full-length transcriptome isoform reconstruction was difficult to perform with existing de novo transcriptome assembly techniques [36]. The de novo transcriptome assembly presents considerable difficulty due to the high sequence similarity among sub-genomes, duplicated genes, and isoforms [37].

The *TI* contigs BLAST results showed that all the contigs were homologous with Kunitz-type *trypsin inhibitor* from various members of the Fabaceae family. In the Kunitz-type family, TI was effective in suppressing the digestion process in various agricultural insects [38]. TI activity in Fabaceae not only inhibits trypsin or chymotrypsin but is sometimes capable of blocking other serine proteases such as subtilisin [39]. The *F. falcata* tree has several insect pests, with *X. festiva* as its main pest. TIs were associated with plants’ resistance against insect pests by inhibiting proteases of the digestive tract, impeding the pests’ development and reproduction [40]. TIs reacted irreversibly with trypsin secreted by the insect gut to form inert-irreversible complexes that inhibit nutrient uptake by the insect and signal the insect to feed less [41]. In another study, overexpression of TI showed antifungal activity, thus not only protecting plants from herbivore insects but also against plant pathogens [42]. In the Fabaceae family, this inhibitor was effective in suppressing metabolic processes in the digestion of agricultural insect pests and was considered more environmentally friendly and more sustainable [38]. There were no Bowman–Birk inhibitor (BBI) type of TI present in the *F. falcata* sequence data in our study, presumably due to the absence of the BBI-type *TI* gene, or the non-expression of the BBI-type *TI* gene. The lack of expression of this gene can be caused by several things, one of which is gene silencing. Silencing at the transcriptional stage was identified by the absence of a transcript of the gene [43].

BLAST results in our study showed that there were similarities in the sequence of AAI in *F. falcata* with AAI chain B (AAI), AAI1, and AAI2 which belong to the lectin class, and CLAI which belongs to the legumin class plant lectin-like amylase inhibitors, AAI1 and AAI2, which mainly exert different inhibitory properties against different types of amylase, while AAI1 has been shown to inhibit mammalian and two insect amylases and AAI2 can only inhibit one insect amylases [44]. Meanwhile, legumin activity against α-amylase was first detected in chickpeas that were able to inhibit α-amylase activity from plants and mammals but were unable to inhibit α-amylase activity from fungi and bacteria [45]. AAI1’s effect on insect larvae was by causing larvae death in the first or second instar; meanwhile, AAI2 resulted in delayed maturation of the larvae [46]. The insect digests starch by secreting α-amylase, which hydrolyzes starch and other related polysaccharides by randomly cleaving internal α-1,4-glucosidic linkages [20]. The inhibitory activity of the AAI against α-amylases was created because of the structure of the inhibitor, which resembled the substrates for α-amylase and therefore binds to the catalytic site of the amylase enzyme [47].

The isoforms found in this study were differentially expressed, with the downregulated isoforms having higher abundance than the upregulated ones. Those various expressions indicated that in those genes, some isoforms acted independently during the larval wounding [48]. The expression of some genes increased due to the larvae’s wounding activity. Meanwhile, the downregulation of *AAI* in non-infested plants, described in another study in persimmon plants [49], probably happened due to the lower starch content. The results of our study showed that the non-infected *F. falcata* had a lower amount of *AAI* expressed. This finding is probably caused by the insect’s larval behavior that has evolved a sensory system for the detection of host cues about the nutritional content [50].

The expression produced by RT-PCR validated the DEG results from RNA-Seq analysis in our study. Both *TI* and *AAI* were mostly expressed in an upregulated way in *X. festiva*-infested samples. These findings indicated that the expression of *TI* and *AAI* in *F. falcata* was induced by the wounding process by *X. festiva* larvae. These findings were supported by a previous study in Poplar [51], in which the Kunitz-type *TI* was strongly induced by wounding and herbivore activity. Another study showed that the activity of NaKTI2 (Kunitz-type *trypsin inhibitor* from Nicotiana attenuata) was highly induced by oral secretions of the herbivore [52]. For *AAI,* it was also identified that the wounding caused by the larvae’ activities increased the level of amylase inhibitor in *Cajanus cajan* [53]. The induction in the host tree also reportedly had a significant impact on lowering the amount of larvae of Trichoplusia feeding on *Brassica rapa* [54].

The insignificant expression of *TI*, as confirmed by the *t*-test in this study, might be caused by the decreased response of the plants due to the prolonged time after initial infestation. Since the infested samples were collected by observing whether there were already burrowing holes and wood leftovers from larvae present, the initial time of the infestations was unknown. Prolonged time after initial infestation could lower the expression of *TI*, according to a former study in tobacco [55]. Another study [56] examined the results after induction by various herbivore attacks in black poplar, but the activity of the *TI* did not correlate with the severity of the damage caused by the herbivores. In another study, *AAI* was also increased by the induction of Lepidoptera larvae in amaranth, but was different from *TI*, which was also able to be induced by salt and water stress, although mechanical human-made wounding failed to induce both inhibitors [16]. The activity of isoforms of *AAI* was affected by pH, temperature, incubation time, and the presence of particular ions [57].

## 4. Materials and Methods

### 4.1. Trypsin Inhibitor and α-Amylase Inhibitor Sequences Alignment and Analysis

**Figure 8:**
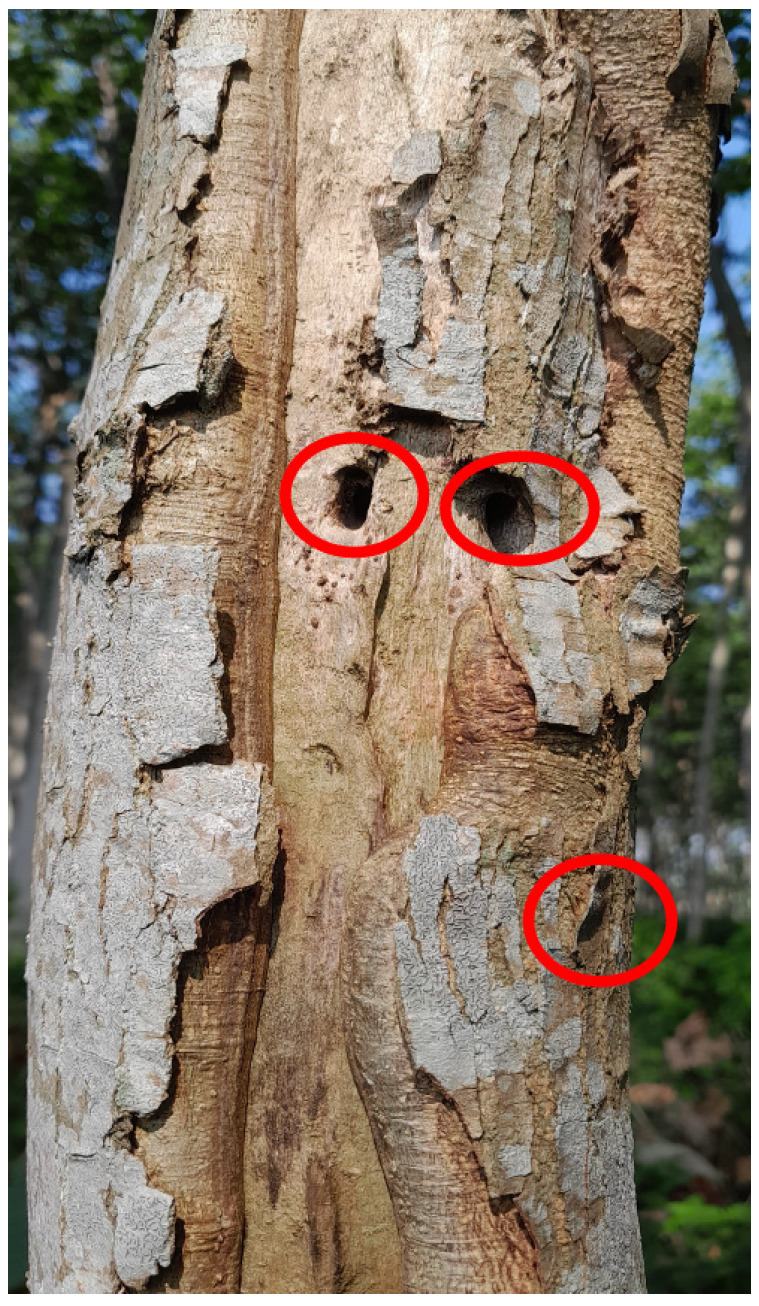
*F. falcata* tree with X. *festiva* burrowing hole (red circle).

The transcriptome sequences from both susceptible and resistant samples were developed from one pair of supposedly resistant and susceptible mature trees grown in a private garden, Bogor, West Java, Indonesia. The resistant and susceptible trees were selected by observing the trees, of which the susceptible ones had one or more *X. festiva* burrowing holes (Figure 8), while the resistant ones had none of them. The presence of the holes indicated that the larvae had successfully overcome the tree’s defenses. The tree pairs were grown in the same plot and separated by a 5 m distance, eliminating possible different environmental factors affecting the larval infestation. The sample collection from susceptible trees considered the burrowing hole conditions, such as the presence of the larvae leftover, which indicates the hole was formed recently. The trees used as samples were planted at the same time, were of the same age, and started to become infested at 3 years old. Total RNA was extracted from the wood tissue close to the bark of the infested sample, which was collected as close as possible to the burrowing hole.

The RNA isolation was performed using Total RNA Mini Kit (Plant) (GeneAid, New Taipei City, Taiwan R.O.C.) with modification using 2 reactions for one sample extracted and adding 26% polyvinylpyrrolidone (PVP) in each tube. A total of 100 mg of wood was ground using mortar with liquid nitrogen and then incubated at 60 °C in the lysis buffer with added PVP and β-Mercaptoethanol. The samples were then centrifuged and filtered to remove the remaining cell debris. The filtered column was washed and then incubated in nuclease-free water for 2 min at room temperature for RNA elution.

The RNA was sequenced using BGI-Seq 500. The RNA-seq protocol consisted of sample preparation, sample QC, library construction, library QC, sequencing, sequencing QC, raw data production, and raw data QC. One of each type of sample was used in the Next-generation sequencing (NGS) process. The raw sequences were uploaded to the database with accession number DRA008389, and the assembled sample is available at https://doi.org/10.6084/m9.figshare.14058458.v1 [25]. The annotated sample was filtered to find the predictive *TI* and *AAI* sequences. The filtered contigs were then trimmed in accordance to their hits in Basic Local Alignment Search Tools (BLAST) results and then aligned using Geneious Prime v.2020.1.2 (https://www.geneious.com, accessed on 5 July 2021) using MUSCLE (Multiple Sequence Comparison by Log-Expectation) algorithm with 10 maximum iterations, kmer value 4_6, and clustering method Unweighted Pair Group Method using Arithmetic Mean (UPGMB). The phylogenetic tree was also generated using Geneious Prime v.2020.1.2 with the Neighbor-Joining method. In performing the alignment and tree construction, we used a sequence of *TI* from soybean (*Glycine max*) with accession number AF128268.1 and *AAI* from the common bean (*Phaseolus vulgaris*) with accession number U10348.1 from GenBank, which were also used as the outgroup for phylogenetic tree development. Those two species were selected because of their similarity with *F. falcata*.

### 4.2. Whole Genome Sequencing (WGS) Analysis

DNA for WGS analysis was extracted from the leaves of a non-infested tree. The sequencing process was conducted with Illumina NovaSeq 6000 by Novogene AIT, Helios, Singapore. The raw data was collected and then analyzed using FASTQC [58] to check the quality of the sequences and filter low-quality data. The filtered sequences were then assembled using Ray v.3 [59] software on the MASER [60] platform. The assembled genome draft was then analyzed using BUSCO v3.0.2 [61] to check the quality and completeness of the data using complete and single copy, complete and duplicated, fragmented, and missing BUSCO criteria. The assembled genome was annotated using annotation after assembling tools in MASER with databases from UniProt and NR NCBI to discover the functional genes in the genome. The sequences that contained the *AAI* and *TI* genes were extracted using Samtools v1.10 [62]. Drafts of genome sequences that contained *TI* and *AAI* aligned with the contigs of *TI-* and *AAI*-containing transcriptomes using Geneious Prime v.2020.1.2 to predict the DNA that transcribes the *TI* and *AAI* genes.

### 4.3. Differentially Expressed Gene (DEG) Analysis

The expression of genes was analyzed by counting the number of reads mapped into the assembled transcriptome using eXpress software v.1.5.1 [63] in counts per million (CPM) [64]. The differentially expressed genes were counted and then analyzed using the edgeR package in R statistical software v.4.2.3 [65]. The DEGs result was then merged with the results of functional annotation from the SwissProt database, with only the TI and AAI sequences. The criterion for determining DEG is a log-fold change (logFC) value of ≥2 and a *p*-value of <0.05. LogFC value ≥ 2 means that the logCPM expression of the gene was ≥2 times higher or lower in the resistant type.

### 4.4. Primer Design for Gene Expression Analysis Using Real Time-Polymerase Chain Reaction (RT-PCR)

The candidate sequences of TI and AAI were then used to generate primers for RT-PCR. Primer design was conducted using Primer3 software [66] with product size ranging between 80 and 150 base pairs (bp), Guanine and Cytosine (GC) content range 45–55%, primer length 17–25 base pairs, and melting temperature (Tm°) range 60–65 °C. We also generated an Actin encoding primer as the housekeeping gene in this study. The data produced more than one sequence for *AAI* and *TI.* Therefore, we developed several primers to test. The designed primers for *TI* and *AAI* are presented in Supplementary Table S1.

The samples used in the RT-PCR process were collected from the same community forest in Bogor, West Java, as the sampling for RNA-seq (−6.55007143, 106.5741218). The wood tissues were collected from 3 resistant and 3 infested *F. falcata* trees. The time after initial infestation in the infested trees was unknown. The paired trees (resistant and infested) were very closely planted at a distance of 3 m from each other. The wood tissue from the infested tree was collected from part of the stem around the larvae’s burrowing hole, while that from the resistant tree was from the healthy stem. The wood was then submerged in Invitrogen RNA-later Stabilization Solution (ThermoFisher, Waltham, MA, USA) until extracted. RNA extraction was performed using Total RNA Mini Kit (Plant) (GeneAid, New Taipei City, Taiwan R.O.C.) with modification using 2 reactions for one sample extracted and adding 26% polyvinylpyrrolidone in each tube. The quality and quantity of extracted RNA were checked with electrophoresis and Nanophotometer (Implen, Munich, Germany). The DNA was then converted to cDNA with ReverTra Ace™ qPCR RT Kit (Toyobo, Osaka, Japan) and stored until RT-PCR analysis.

The RT-PCR process was performed using Applied Biosystem StepOne™Plus System (Termofisher, Waltham, MA, USA). The RT-PCR reaction contained ~10 ng cDNA as template, 400 nM of each forward and reverse primer, and 5 µL SensiFAST™ SYBR^®^ Hi-ROX One-Step mix (2×) (Bioline, London, UK). The RT-PCR process was started with activation of Ampli-Taq at 95 °C for 3 min, followed by 40 cycles of denaturation at 95 °C, annealing, and extension at 72 °C. The RT-PCR results were also electrophoresed in 2% agarose gel. RT-PCR was conducted with 3 biological replicates for each sample.

The relative expression of targeted genes was then analyzed using the quantification method relative to the comparison of Cycle threshold (Ct) values (2^−ΔΔCT^) [67]:

∆Ct_P_ = Ct_GT_ − Ct_HG_;

∆Ct_K_ = Ct_GT_ − Ct_HG_;

∆∆Ct = ∆Ct_P_ − ∆Ct_K_

Expression = 2^−∆∆Ct^

Notes: Ct_GT_: Ct value of targeted gene; Ct_HG_: Ct value of *Housekeeping gene*; ∆Ct_P_: Ct value of treated sample; ∆Ct_K_: Ct value of control sample; ∆∆Ct: difference of Ct value between treated and control sample.

Statistical analysis was performed on the expression value of resistant and infested samples on both *TI* and *AAI* genes using paired *T*-test.

## 5. Conclusions

Isoforms of *TI* and *AAI* encoding genes found in this research indicated that there was various alternative splicing in those genes and indicated that the expression of the same gene at the same time could differ due to the different functions of each isoform. The isoforms of the *TI* and *AAI* genes are possibly formed by a single copy of DNA for each gene. RT-PCR results confirmed the majority of DEG analysis, which indicates that both inhibitors’ activity was heavily induced by the *X. festiva* wounding and gut enzyme. The expression in infested samples of *AAI* was significantly upregulated 2.44 times on average, while the *TI* gene insignificantly increased 0.78 times on average. Findings from this research could be the foundation for future studies on how to induce *TI* and *AAI* activity to strengthen *F. falcata* resistance against *X. festiva* infestation or other insect pests. The findings from this research are also important in developing markers for selecting *X. festiva*-resistant trees. Further study into the interaction between both inhibitor activity and other signaling-related genes also needs to be pursued.

## Figures and Tables

**Table 1:** Contigs of *TI* and *AAI* found in *Falcataria falcata* wood transcriptomes.

No.	Contig Name	Hits	Accession Number
*Trypsin Inhibitor*			
1	Cluster-42052.303167_TRINITY_DN172546_c1_g2_i2	1023–778	P32733.1
2	Cluster-42052.306924_TRINITY_DN178415_c6_g1_i1	569–63	4J2Y_A
3	Cluster-42052.247290_TRINITY_DN178415_c6_g6_i1	2–454	P24924.2
4	Cluster-42052.206862_TRINITY_DN181734_c1_g2_i3	1006–575	P83036.2
5	Cluster-42052.195413_TRINITY_DN177358_c0_g2_i2	1218–697	AFG28551.1
6	Cluster-42052.169979_Contig18349	2423–1929	AFG28551.1
7	Cluster-42052.253142_TRINITY_DN169753_c0_g1_i1	690–346	P83036.2
8	Cluster-42052.207278_TRINITY_DN191341_c4_g2_i4	722–186	AFG28551.1
9	Cluster-42052.155280_TRINITY_DN182112_c0_g2_i2	704–312	AFG28551.1
10	Cluster-42052.175330_Contig18348	1857–1363	AFG28551.1
11	Cluster-42052.266424_TRINITY_DN160773_c0_g1_i1	443–994	P24924.2
12	Cluster-42052.180501_TRINITY_DN171608_c0_g1_i1	703–197	P83036.2
13	Cluster-42052.15862_TRINITY_DN183193_c0_g1_i6	585–869	P09941.1
14	Cluster-42052.179981_TRINITY_DN168277_c1_g1_i2	507–683	AAB26177.1
15	Cluster-42052.61489_Contig29962	430–978	P83036.2
16	Cluster-42052.228242_TRINITY_DN169156_c0_g1_i1	426–884	P24924.2
17	Cluster-42052.208643_TRINITY_DN175236_c0_g1_i2	238–101	P32733.1
18	Cluster-42052.77508_TRINITY_DN181117_c0_g1_i2	380–93	AFG28551.1
19	Cluster-42052.264216_TRINITY_DN129174_c0_g1_i1	768–643	AFG28551.1
α-amylase Inhibitor			
1	Cluster-42052.208011_TRINITY_DN177772_c6_g3_i6	869–1558	1VIW_B
2	Cluster-42052.237331_TRINITY_DN186379_c1_g2_i1	615–1154	1VIW_B
3	Cluster-42052.144807_TRINITY_DN174608_c1_g1_i1	345–1019	1VIW_B
4	Cluster-42052.138754_TRINITY_DN181383_c6_g1_i1	551–1126	P02873
5	Cluster-42052.162634_TRINITY_DN181383_c6_g1_i2	551–1126	P02873
6	Cluster-42052.188408_TRINITY_DN182603_c3_g1_i1	436–1053	1VIW_B
7	Cluster-42052.155248_Contig10939	1–210	1VIW_B
8	Cluster-42052.100030_TRINITY_DN177479_c0_g1_i1	1899–1267	P02873
9	Cluster-42052.216188_TRINITY_DN186223_c4_g4_i5	3177–3377	1VIW_B
10	Cluster-42052.319012_TRINITY_DN172082_c0_g1_i3	738–1442	1VIW_B
11	Cluster-42052.201855_Contig18100	104–808	P02873
12	Cluster-42052.195476_TRINITY_DN164741_c0_g2_i1	498–1067	1VIW_B
13	Cluster-42052.177708_TRINITY_DN172458_c0_g1_i1	543–55	Q9SMJ4
14	Cluster-42052.235439_TRINITY_DN180890_c2_g1_i2	320–655	Q9SMJ4
15	Cluster-42052.341257_TRINITY_DN180890_c2_g1_i1	492–869	Q9SMJ4
16	Cluster-42052.245145_TRINITY_DN180307_c1_g4_i3	478–1164	1VIW_B
17	Cluster-42052.216103_TRINITY_DN186574_c0_g1_i2	638–1312	1VIW_B
18	Cluster-42052.420818_TRINITY_DN182603_c3_g1_i4	490–1107	1VIW_B
19	Cluster-42052.179748_TRINITY_DN185634_c1_g3_i3	571–1143	P02873
20	Cluster-42052.245310_TRINITY_DN185634_c1_g3_i4	511–1086	Q41114
21	Cluster-42052.169697_Contig9179	754–53	P02873
22	Cluster-42052.182397_TRINITY_DN175336_c0_g1_i2	677–1312	P02873
23	Cluster-42052.207959_TRINITY_DN183003_c2_g5_i3	293–541	P02873
24	Cluster-42052.189890_TRINITY_DN184034_c1_g5_i1	167–562	P02873
25	Cluster-42052.218695_Contig38867	1279–551	Q9SMJ4
26	Cluster-42052.207925_TRINITY_DN173791_c0_g1_i3	1–402	P02873
27	Cluster-42052.24436_Contig7754	570–770	1VIW_B
28	Cluster-42052.123152_TRINITY_DN184034_c1_g3_i1	245–36	1VIW_B
29	Cluster-42052.292956_TRINITY_DN176191_c0_g1_i1	457–110	P02873

**Table 2:** DEG results for TI and AAI in *F. falcata*.

TI	Sequence	logFC	*p*-Value	Name
	Cluster-42052.169979_Contig18349	−11.472	1.15 × 10^−25^	AFG28551.1 Kunitz *trypsin inhibitor*
	Cluster-42052.15862_TRINITY_DN183193_c0_g1_i6	−10.1102	8.55 × 10^−17^	sp|P09941.1| *trypsin inhibitor* DE5 alpha chain
	Cluster-42052.179981_TRINITY_DN168277_c1_g1_i2	−9.64578	4.26 × 10^−14^	AAB26177.1 Kunitz-type *trypsin inhibitor* A chain,
	Cluster-42052.146119_TRINITY_DN173153_c0_g3_i1	−9.58357	9.45 × 10^−14^	AFG28551.1 Kunitz *trypsin inhibitor*, partial
	Cluster-42052.231745_TRINITY_DN191341_c4_g2_i3	−9.27791	3.82 × 10^−12^	sp|C0HKQ3.1| Kunitz-type *trypsin inhibitor* IVTI
	Cluster-42052.155280_TRINITY_DN182112_c0_g2_i2	−8.10613	3.57 × 10^−22^	AFG28551.1 Kunitz *trypsin inhibitor*,
	Cluster-42052.175330_Contig18348	−7.14287	9.21 × 10^−22^	AFG28551.1 Kunitz *trypsin inhibitor*,
	Cluster-42052.208643_TRINITY_DN175236_c0_g1_i2	−5.77413	1.75 × 10^−13^	sp|P32733.1|Kunitz-type *trypsin inhibitor* alpha chain
	Cluster-42052.61489_Contig29962	−5.41235	2.84 × 10^−13^	sp|P83036.2| *trypsin inhibitor*
	Cluster-42052.206862_TRINITY_DN181734_c1_g2_i3	−5.18019	9.31 × 10^−22^	sp|P83036.2| Full = *trypsin inhibitor*;
	Cluster-42052.195413_TRINITY_DN177358_c0_g2_i2	−4.90233	6.39 × 10^−20^	AFG28551.1 Kunitz *trypsin inhibitor*, partial
	Cluster-42052.228242_TRINITY_DN169156_c0_g1_i1	−4.81848	3.13 × 10^−10^	sp|P24924.2| *trypsin inhibitor*
	Cluster-42052.180501_TRINITY_DN171608_c0_g1_i1	−4.17532	8.19 × 10^−12^	sp|P83036.2|*trypsin inhibitor*
	Cluster-42052.207278_TRINITY_DN191341_c4_g2_i4	−3.68564	3.05 × 10^−12^	AFG28551.1 Kunitz *trypsin inhibitor*
	Cluster-42052.77508_TRINITY_DN181117_c0_g1_i2	−3.32757	6.14 × 10^−7^	AFG28551.1 Kunitz *trypsin inhibitor*
	Cluster-42052.264216_TRINITY_DN129174_c0_g1_i1	−1.20859	0.033664	AFG28551.1 Kunitz *trypsin inhibitor*
	Cluster-42052.266424_TRINITY_DN160773_c0_g1_i1	−0.54328	0.279542	sp|P24924.2| *trypsin inhibitor*
	Cluster-42052.253142_TRINITY_DN169753_c0_g1_i1	−0.14329	0.76744	sp|P83036.2| *trypsin inhibitor*
	Cluster-42052.247290_TRINITY_DN178415_c6_g6_i1	1.764728	0.00016	sp|P24924.2|*trypsin inhibitor*
	Cluster-42052.306924_TRINITY_DN178415_c6_g1_i1	3.478816	1.12 × 10^−13^	pdb|4J2Y|A Chain A, *trypsin inhibitor*
	Cluster-42052.303167_TRINITY_DN172546_c1_g2_i2	4.027332	9.26 × 10^−18^	sp|P32733.1| Kunitz-type *trypsin inhibitor* alpha chain
AAI	Sequence	logFC	*p*-Value	Name
	Cluster-42052.100030_TRINITY_DN177479_c0_g1_i1	2.01585	3.416676	sp|P02873.1| Alpha-amylase inhibitor 1
	Cluster-42052.235439_TRINITY_DN180890_c2_g1_i2	1.47664	1.707336	sp|Q9SMJ4.1| Alpha-amylase inhibitor
	Cluster-42052.341257_TRINITY_DN180890_c2_g1_i1	1.04942	1.408281	sp|Q9SMJ4.1| Alpha-amylase inhibitor
	Cluster-42052.216188_TRINITY_DN186223_c4_g4_i5	1.02626	3.460806	pdb|1VIW|B Chain B, ALPHA-AMYLASE-INHIBITOR
	Cluster-42052.155248_Contig10939	0.97774	3.686339	pdb|1VIW|B Chain B, ALPHA-AMYLASE-INHIBITOR
	Cluster-42052.144807_TRINITY_DN174608_c1_g1_i1	0.76538	4.318647	pdb|1VIW|B Chain B, ALPHA-AMYLASE-INHIBITOR
	Cluster-42052.208011_TRINITY_DN177772_c6_g3_i6	0.50414	4.926196	pdb|1VIW|B Chain B, ALPHA-AMYLASE-INHIBITOR
	Cluster-42052.162634_TRINITY_DN181383_c6_g1_i2	0.37354	4.001451	sp|P02873.1| Alpha-amylase inhibitor 1; Short = Alpha-AI-1;
	Cluster-42052.420818_TRINITY_DN182603_c3_g1_i4	0.31528	1.416495	pdb|1VIW|B Chain B, ALPHA-AMYLASE-INHIBITOR
	Cluster-42052.138754_TRINITY_DN181383_c6_g1_i1	0.22861	4.274507	sp|P02873.1| Alpha-amylase inhibitor 1
	Cluster-42052.237331_TRINITY_DN186379_c1_g2_i1	0.09506	4.693314	pdb|1VIW|B Chain B, ALPHA-AMYLASE-INHIBITOR
	Cluster-42052.177708_TRINITY_DN172458_c0_g1_i1	−0.17696	2.1322	sp|Q9SMJ4.1| Alpha-amylase inhibitor
	Cluster-42052.319012_TRINITY_DN172082_c0_g1_i3	−0.21007	3.530725	pdb|1VIW|B Chain B, ALPHA-AMYLASE-INHIBITOR
	Cluster-42052.147883_Contig6928	−0.25073	4.212243	sp|P02873.1| Alpha-amylase inhibitor 1
	Cluster-42052.189890_TRINITY_DN184034_c1_g5_i1	−0.38222	0.493207	sp|P02873.1| Alpha-amylase inhibitor 1
	Cluster-42052.188408_TRINITY_DN182603_c3_g1_i1	−0.60056	3.938534	pdb|1VIW|B Chain B, ALPHA-AMYLASE-INHIBITOR
	Cluster-42052.209935_TRINITY_DN185927_c2_g3_i1	−0.60486	3.325443	sp|P84708.1| Chitinolytic alpha-amylase inhibitor
	Cluster-42052.24436_Contig7754	−0.79325	−0.016	pdb|1VIW|B Chain B, ALPHA-AMYLASE-INHIBITOR
	Cluster-42052.123152_TRINITY_DN184034_c1_g3_i1	−0.8583	0.428841	pdb|1VIW|B Chain B, ALPHA-AMYLASE-INHIBITOR
	Cluster-42052.169697_Contig9179	−1.38486	1.437683	sp|P02873.1| Alpha-amylase inhibitor 1
	Cluster-42052.216103_TRINITY_DN186574_c0_g1_i2	−1.64042	2.126177	pdb|1VIW|B Chain B, ALPHA-AMYLASE-INHIBITOR
	Cluster-42052.195476_TRINITY_DN164741_c0_g2_i1	−1.72911	2.658958	pdb|1VIW|B Chain B, ALPHA-AMYLASE-INHIBITOR
	Cluster-42052.201855_Contig18100	−2.01150	2.602573	sp|P02873.1| Alpha-amylase inhibitor 1
	Cluster-42052.182397_TRINITY_DN175336_c0_g1_i2	−2.11116	1.290499	sp|P02873.1| Alpha-amylase inhibitor 1
	Cluster-42052.359247_TRINITY_DN178532_c0_g1_i2	−2.11682	1.589345	sp|P84708.1| Chitinolytic alpha-amylase inhibitor
	Cluster-42052.206287_TRINITY_DN186880_c2_g2_i2	−3.26811	7.775568	sp|P84708.1| Chitinolytic alpha-amylase inhibitor PvCAI
	Cluster-42052.218695_Contig38867	−3.79574	0.394497	sp|Q9SMJ4.1| Alpha-amylase inhibitor
	Cluster-42052.207925_TRINITY_DN173791_c0_g1_i3	−3.85575	0.450129	sp|P02873.1| Alpha-amylase inhibitor 1
	Cluster-42052.238154_Contig19707	−5.90505	3.19328	pdb|1VIW|B Chain B, ALPHA-AMYLASE-INHIBITOR
	Cluster-42052.292956_TRINITY_DN176191_c0_g1_i1	−9.35441	0.579165	sp|P02873.1| Alpha-amylase inhibitor 1
	Cluster-42052.207959_TRINITY_DN183003_c2_g5_i3	−9.54055	0.769758	sp|P02873.1| Alpha-amylase inhibitor 1
	Cluster-42052.245310_TRINITY_DN185634_c1_g3_i4	−10.2767	1.526612	sp|Q41114.1| Alpha-amylase inhibitor 2
	Cluster-42052.179748_TRINITY_DN185634_c1_g3_i3	−10.4377	1.69216	sp|P02873.1| Alpha-amylase inhibitor 1
	Cluster-42052.245145_TRINITY_DN180307_c1_g4_i3	−10.7524	2.015186	pdb|1VIW|B Chain B, ALPHA-AMYLASE-INHIBITOR

**Table 3:** Primer sequences that successfully produce a specific band.

Primer	Sequence	Tm (°C)	Tm (°C)
TI			
Forward	GACAGGAAACGAAACTTGCCC	GACAGGAAACGAAACTTGCCC	61.3
Reverse	ACGAAATTTTCCATGGCAAGCC	ACGAAATTTTCCATGGCAAGCC	60.3
AAI			
Forward	AGGAAACGAAGGAAAG CGCGG	AGGAAACGAAGGAAAG CGCGG	61.3
Reverse	TTGCCGTTTCCTCCGAT TCC	TTGCCGTTTCCTCCGAT TCC	60.5
Actin			
Forward	TTGACTGCGCTTCATCACCC	TTGACTGCGCTTCATCACCC	60.5
Reverse	GGCTGGTTTTGCTGGTGATG	GGCTGGTTTTGCTGGTGATG	60.5

## Data Availability

The transcriptome data used in this research are available for access at https://doi.org/10.6084/m9.figshare.14058458.v1. Raw data of the Genome sequence available at DDBJ with accession number DRA012508 (https://ddbj.nig.ac.jp/search/entry/sra-submission/DRA012508, accessed on 31 July 2021).

## Supplementary Materials

The following supporting information can be downloaded at: https://www.mdpi.com/article/10.3390/plants14172750/s1. Table S1. list of primers for *TI* and *AAI* genes of *F. falcata*.
