# Supplementary material for: Multi-Transcripts and Expressions of Trypsin Inhibitor and α-Amylase Inhibitor Genes of Sengon (Falcataria falcata) Against Xystrocera festiva Stem Borer Infestation
Source: Plants (Basel). 2025 Sep 3;14(17):2750. doi: 10.3390/plants14172750 (PMC12430205; doi:10.3390/plants14172750)
Supplement: Supplementary file 1 [file plants-14-02750-s001.zip › plants-3759140-supplementary.pdf]

Supplementary Table S1. List of primers for TI and AAI genes of *F. falcata.*

Tm (°C)	Primer sequence	Primer	Primer	Gene
60.04	TCTACGTCGGCCAGGAAAAC	Forwad	1	TI
60.04	TCACGACGTTGTCTTCTCCG	Reverse		
60.27	ACCTTCAATTGCCTGACCTCC	Forwad	2	
60.32	TCTCTTTGCCATCACCACGG	Reverse		
61.22	TCGTTTCCTGTCGCAGCAAG	Forwad	3	
60.41	TCTCTTTGCCTTCATCGCCTC	Reverse		
60.00	GACAGGAAACGAAACTTGCCC	Forwad	4	
60.61	ACGAAATTTTCCATGGCAAGCC	Reverse		
60.89	TCTTGCACAACGACATGGGG	Forwad	5	
60.18	TACATCTTGCCGGCCATCTG	Reverse		
60.25	TCTTCAGCAGCCTCCAAACC	Forwad	6	
60.32	TGACATTCTCACCGGCCAAG	Reverse		
60.11	CAATTCCTCACCGTTTGCCG	Forwad	7	
60.03	TCTCCGATGACAAAGACACAGG	Reverse		
60.43	CGAGGAAGAGACTGAAGATTGGG	Forwad	1	AAI
60.11	TTCCCTCCAAACCCAACCAC	Reverse		
60.61	AGGAAACGAAGGAAAGCGAGG	Forwad	2	
60.68	TTGCCGTTTCCTCCGATTCC	Reverse		
61.12	GTAGGTGACTCTCCCAATGCTG	Forwad	3	
60.29	GAAGGAGATGCAGACTACGACG	Reverse		
60.06	AGGAGGCCATTGGAAGTGATG	Forwad	4	
60.25	GGCGTTGGTCCTTTTGTTGG	Reverse		
63.08	CGGGCAATACTCTCCTCAAGTC	Forwad	5	
60.18	CAGACGGAGCTATAGTGAAGGC	Reverse		
60.27	ATCTTCCGTTGGTTGGTGAGG	Forwad	6	
60.22	GACCAACAACTTGAACACGACC	Reverse		
60.12	ACCAGGAACATGAGCAGTAGATG	Forwad	7	
60.07	CAAACGCAGGAAAAGAGGTCG	Reverse		
